# Identification of Comorbidities, Genomic Associations, and Molecular Mechanisms for COVID-19 Using Bioinformatics Approaches

**DOI:** 10.1155/2023/6996307

**Published:** 2023-01-11

**Authors:** Shudeb Babu Sen Omit, Salma Akhter, Humayan Kabir Rana, A. R. M. Mahamudul Hasan Rana, Nitun Kumar Podder, Mahmudul Islam Rakib, Ashadun Nobi

**Affiliations:** ^1^Department of Computer Science and Telecommunication Engineering, Noakhali Science and Technology University, Noakhali 3814, Bangladesh; ^2^Department of Environmental Science and Disaster Management, Noakhali Science and Technology University, Noakhali 3814, Bangladesh; ^3^Department of Computer Science and Engineering, Green University of Bangladesh, Dhaka 1207, Bangladesh; ^4^Department of Computer Science and Engineering, Khulna University of Engineering & Technology, Khulna 9203, Bangladesh

## Abstract

Several studies have been done to identify comorbidities of COVID-19. In this work, we developed an analytical bioinformatics framework to reveal COVID-19 comorbidities, their genomic associations, and molecular mechanisms accomplishing transcriptomic analyses of the RNA-seq datasets provided by the Gene Expression Omnibus (GEO) database, where normal and infected tissues were evaluated. Using the framework, we identified 27 COVID-19 correlated diseases out of 7,092 collected diseases. Analyzing clinical and epidemiological research, we noticed that our identified 27 diseases are associated with COVID-19, where hypertension, diabetes, obesity, and lung cancer are observed several times in COVID-19 patients. Therefore, we selected the above four diseases and performed assorted analyses to demonstrate the association between COVID-19 and hypertension, diabetes, obesity, and lung cancer as comorbidities. We investigated genomic associations with the cross-comparative analysis and Jaccard's similarity index, identifying shared differentially expressed genes (DEGs) and linking DEGs of COVID-19 and the comorbidities, in which we identified hypertension as the most associated illness. We also revealed molecular mechanisms by identifying statistically significant ten pathways and ten ontologies. Moreover, to understand cellular physiology, we did protein-protein interaction (PPI) analyses among the comorbidities and COVID-19. We also used the degree centrality method and identified ten biomarker hub proteins (IL1B, CXCL8, FN1, MMP9, CXCL10, IL1A, IRF7, VWF, CXCL9, and ISG15) that associate COVID-19 with the comorbidities. Finally, we validated our findings by searching the published literature. Thus, our analytical approach elicited interconnections between COVID-19 and the aforementioned comorbidities in terms of remarkable DEGs, pathways, ontologies, PPI, and biomarker hub proteins.

## 1. Introduction

The coronavirus disease 2019 (COVID-19) is an infectious disease that was caused by severe acute respiratory syndrome coronavirus 2 (SARS-CoV-2). This disease was first reported in December 2019 in Wuhan, Hubei Province, China. At that time, this virus was transmitted directly from one person to another through direct contact or the droplets from the infected person's respiratory system [1, 2]. Therefore, it spread to almost all the countries in the world very fast, and due to the rapid outbreak of COVID-19, the World Health Organization (WHO) declared it as a global pandemic on 11 March 2020. According to the WHO, there are more than 635 million confirmed cases and almost 6.61 million deaths around the world caused by COVID-19 as of 15 November 2022, where the underlying comorbidities often lead to these deaths [3]. Also, the situation of COVID-19 gets more severe due to the existing comorbid conditions, as morbidity and mortality are associated with multiple chronic conditions [4, 5]. Studies further suggest that COVID-19 also affects the organs and organ systems of the human body, which is also responsible for patient death [6–9]. Hence, we proposed an approach that can ascertain COVID-19's comorbidities, genomic association, and molecular mechanisms in order to reduce the progression of the disease and make the protection, prevention, and treatment policies for COVID-19 patients early.

In order to identify comorbidities associated with COVID-19, numerous studies have been conducted. Aktar et al. performed a meta-analysis on the published available global literature and used supervised machine learning algorithms on clinical cohort data with existing comorbidity information to identify significant comorbidities of COVID-19 [10]. Chakrabarty et al. selected three viral attack receptors and identified proteins associated with those receptors; using the proteins, they collected diseases and named them as comorbidities [11]. Singh et al. performed meta-analysis and coexpression analysis for the correlation pattern in genes and showed the susceptibility of COVID-19 with leukemia, nonalcoholic fatty liver diseases, psoriasis, diabetes, and pulmonary arterial hypertension as comorbidities [12]. Atkins et al. used UK Biobank and identified the true risk factors of COVID-19 from the preexisting diagnosis and hospitalized COVID-19 patients' data [13]. Satu et al. selected breast cancer, colon cancer, kidney cancer, liver cancer, bladder cancer, prostate cancer, thyroid cancer, and lung cancer, revealed the association as well as the interaction between selected cancers and COVID-19, and then marked the cancers as comorbidities [14]. Dolan et al. chose five diseases: kidney disease, liver disease, diabetes, lung disease, and cardiovascular disease as comorbidities from the literature and showed the association of those disorders with COVID-19 [15]. Yang et al. performed a meta-analysis from laboratory confirmed COVID-19 studies; they identified and evaluated the comorbidities and dissemination in COVID-19 patients [16]. Thakur et al. also performed a systematic review and meta-analysis to estimate COVID-19's comorbidities and their prevalence, severity as well as morality related to age, gender, and geographical areas using pooled proportion [17]. We found that the majority of works for the identification of COVID-19 comorbidities are based on meta-analyses without examining gene products, functional associations, and influencing factors. Therefore, we have performed bioinformatics approaches to identify the comorbidities and to evaluate the genomic as well as molecular associations between COVID-19 and the comorbidities.

In this study, we have used the transcriptomic data derived from the blood samples of COVID-19 patients and healthy people. We analyzed the dataset with GREIN [18] tool and detected up and downregulated genes. Using these dysregulated genes, we collected a list of associated diseases from the Gold Benchmark database that is referenced in the following methods and analyses segment. Then, the statistically significant diseases are filtered from the accumulated COVID-19 allied diseases. Next, we enumerated associated genes that are enriched in individual significant diseases, and we ordered the diseases depending on the calculated enriched gene number according to descending sequence. After eliminating the homogeneous illnesses from the top of the ranked list, we selected comorbidities that we considered as consequences. To perform the genomic associations into COVID-19 and the identified comorbidities as well as the inter comorbidities, we used bipartite graphs or networks, in which at least one remarkable overexpressed or underexpressed gene should be shared by COVID-19 and the comorbidities or within the comorbidities. We also utilized the Jaccard similarity index to identify the most prevalent comorbidity among the selected comorbidities. To evaluate the molecular mechanisms, we identified statistically significant pathways and ontologies. We also performed protein-protein interaction analysis and ranked ten influential biomarker hub proteins based on the degree, as discussed in the methods and analyses section in detail. Finally, we confirmed our results through global research that indicates the association of COVID-19 with hypertension, diabetes, obesity, and lung cancer.

## 2. Methods and Analyses

### 2.1. Dataset Collection

For the identification of comorbidities, we collected the SARS-CoV-2 (COVID-19) dataset (accession number GSE150819) from NCBI's (https://www.ncbi.nlm.nih.gov/) public Gene Expression Omnibus (GEO) genomics data repository. The dataset is an RNA-seq dataset prepared from human bronchial epithelial cells of COVID-19 patients and healthy people. GREIN is an interactive web platform that provides options to analyze GEO RNA-seq data for uniform processing [18]. Utilizing GREIN, we got our processed COVID-19 (GSE150819) primary dataset for further analysis. Similarly, we used NCBI and GREIN to collect RNA-seq datasets for hypertension, diabetes, obesity, and lung cancer of accession numbers GSE143953, GSE136053, GSE55008, and GSE60052. We examined several datasets for both COVID-19 and the aforementioned four diseases and discarded most of them as the datasets did not fulfill our criteria because of insufficient sample size, missing control or case samples, absence of gene symbols, incomplete formatting, and duplicate samples. We also rejected the datasets that were not generated from human organisms or *Homo sapiens*. Considering all of the criteria, we selected the gestational hypertension dataset for hypertension which is derived from placenta cells of affected and control samples employing Illumina HiSeq 4000 technology. The dataset for diabetes is produced from peripheral blood mononuclear cells of healthy and diseased samples using Illumina HiSeq 2500 technology. The dataset for obesity is obtained by gene expression profiling and Illumina HiSeq 2000 technology from omental adipose tissues of control and case samples. And, the dataset for lung cancer is generated using Illumina HiSeq 2000 technology from human lung tissues of infected and uninfected samples. The summary of the datasets is presented in Table 1.

### 2.2. Differentially Expressed Gene (DEG) Identification

For the affirmation of COVID-19 comorbidities and their association, we identified differentially expressed genes from COVID-19, hypertension, diabetes, obesity, and lung cancer based on a threshold *P* value and log2 fold change value. The identification of DEGs can be mathematically expressed as follows:

(1) $$\begin{array}{c} {\text{DEGs}}_{\left(i\right)}=\left\{\begin{array}{c} \text{Upregulated},P\text{ value}<0.05\text{ and logFC}>1,\\ \text{Downregulated},P\text{ value}<0.05\text{ and logFC}<-1, \end{array}\right. \end{array}$$

where DEGs_(*i*)_ is the *i*^th^ differentially expressed gene which can be either upregulated or downregulated based on the above equation criteria.

### 2.3. Disease Collection

After selecting the COVID-19 dataset from NCBI and processing it with GREIN, we found our final dataset from where we derived the DEGs assuming Equation (1). Enrichr [24] is a web-based gene-enrichment analysis platform that contains several databases presenting diseases with *P* value, adjusted *P* value, old *P* value, old adjusted *P* value, odds ratio, combined score, and associated genes. Utilizing the identified DEGs of COVID-19, we obtained diseases from the DisGeNET [25] database of EnrichR. Next, we filtered the collected diseases and retained only the statistically significant diseases according to *P* value < 0.05.

### 2.4. COVID-19 Comorbidity Identification and Selection

There are a number of enriched genes in the statistically significant diseases. We counted the enriched genes for each disease individually. After that, we ranked the individual diseases from top to bottom in descending order based on the counted gene number that we calculated before. Then, we considered the first 50 diseases and removed identical diseases from the arranged disease list. Finally, we reviewed the clinical and epidemiological research to select comorbidities based on their existence in COVID-19 patients. After the selection process, following analyses were executed to validate the relationship between COVID-19 and the comorbidities. Again, we also investigated whether the selected comorbidities are interconnected or not.

### 2.5. COVID-19 and Selected Comorbidity Association

Utilizing neighborhood flourished standard and topological approaches, we constructed bipartite graphs or networks for the gene-disease association where the nodes are genes (round shaped) or diseases (octagonal shaped) and validated the correlation among COVID-19 and its selected comorbidities. To participate in the network as well as the association or connection, the diseases (COVID-19 and the comorbidities) should have shared one or more significant DEGs within them [26]. We considered that *D* is a set of diseases, and *G* is a set of DEGs; then, the bipartite graph or the network is determined by whether gene *g* ∈ *G* is affiliated with disease *d* ∈ *D*. If DEGs *G*_*X*_ and *G*_*Y*_ are gradually associated with diseases *D*_*X*_ and *D*_*Y*_, then the duplicated shared DEGs (*n*_XY_^*g*^) for both upregulated and downregulated genes in the diseases can be expressed mathematically as follows:

(2) $$\begin{array}{c} n_{\text{XY}}^{g}=N\;\left(G_{X}\cap G_{Y}\right). \end{array}$$

The common adjacent neighbors and the interaction are recognized by measuring the edge score (*E*) for each node pair through the Jaccard similarity index [26–28] as follows:

(3) $$\begin{array}{c} E\;\left(X,Y\right)=\frac{N\left(G_{X}\cap G_{Y}\right)}{N\;\left(G_{X}\cup G_{Y}\right)}. \end{array}$$

Here, *G* and *E* are the set of nodes and edges separately. Again, in the networks or bipartite graphs, cooccurrence is the number of shared genes.

### 2.6. Molecular Pathway and Gene Ontology Analyses

To understand the molecular level interactions, internal changes of cells and organisms as well as how complex diseases get linked to each other's underlying biological mechanism, pathway analysis is vital. Therefore, we performed pathway analysis to derive the relationship between COVID-19 and hypertension, diabetes, obesity, and lung cancer. We utilized commonly altered DEGs of COVID-19 and each of the selected maladies in KEGG [29, 30], WikiPathways [29, 31], and Reactome [29, 32] databases of Enrichr for pathway analysis. Parameters to select the most significant pathways are statistical *P* value < 0.05 and literature searches.

To convey gene activities, their correlation, and the mechanisms that influence diseases, gene ontology is essential. Therefore, we executed gene ontology analysis to understand the correlation between COVID-19 and each of the comorbidities. We used the commonly altered DEGs of COVID-19 and hypertension, diabetes, obesity, and lung cancer in GO Biological Process [33] and GO Cellular Component [34] databases of Enrichr and collected ontologies. We employed the same parameters for the significant ontologies identification that we used in the pathway analysis.

### 2.7. Protein-Protein Interaction (PPI) Analyses and Biomarker Hub Protein Identification

Mathematical and graphical illustration for the structural organization of proteins in a cellular unit is known as protein-protein interaction. To understand cellular/molecular physiology formed by complex biochemical functions and also for drug development, PPI is crucial [26, 34]. Therefore, we performed PPI analysis using the commonly altered DEGs between COVID-19 and its comorbidities for the possible linkage among them, utilizing the STRING (https://string-db.org/) database. We used the medium confidence score of 0.4 as a threshold for the PPI network construction and Cytoscape [35] for the topological analysis where the disconnected proteins are removed. In PPI, the distance (DS) between a pair of protein *i* and protein *j* is mathematically expressed as follows:

(4) $$\begin{array}{c} \text{DS}\left(i,j\right)=\frac{2\left|N_{i}\cap N_{j}\right|}{\left|N_{i}\right|\cup \left|N_{j}\right|}, \end{array}$$

where *N*_*i*_ and *N*_*j*_ denote a set of neighbors *i* and *j*.

Hub proteins are proteins that are connected to several other proteins in a considerable number. They are potential biomarkers because during the development of COVID-19 and its comorbidities, hub proteins play a part in signal transmission as well as they may lead to effective remedy targets [36]. We identified hub proteins from the proteins of the STRING database using all overlapping DEGs of COVID-19 and the comorbidities. We considered only highly connected proteins during hub protein identification. The degree centrality (DC) is applied for our biomarker hub proteins, and in this regard, cytoHubba plugin [37] is used. Degree centrality can be expressed as follows:

(5) $$\begin{array}{c} \text{DC}\left(v\right)=\underset{i\in G}{\sum }\frac{a_{\text{vi}}}{N-1}, \end{array}$$

where DC is the degree centrality of a node *v*, *N* is the total number of nodes in the network  *G* and *a*_vi_ is the direct connection between nodes *v* and *i*.

### 2.8. Comorbidity Interrelationship Analysis

**Figure 1:**
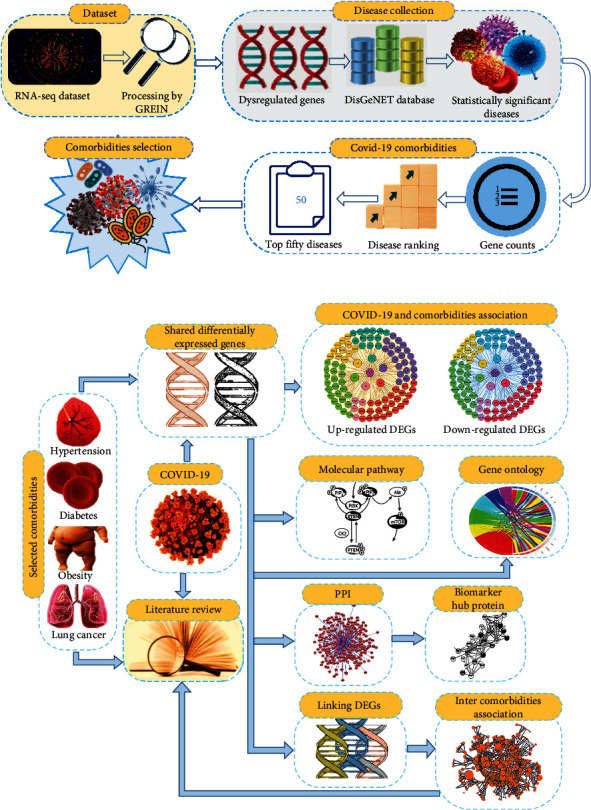
Workflow of this research methodology. (a) Analytical approach for comorbidity identification. (b) Analytical approach for comorbidity genomic associations and molecular mechanisms with COVID-19.

We also examined the interrelationships between the selected comorbidities. For that, we considered the comorbidities' frequent dysregulated genes or DEGs with COVID-19. Using Equations (2) and (3), we identified the overlapped linking DEGs along with the gene-comorbidity association network among the comorbidities themselves. Moreover, we searched worldwide published scientific articles to figure out how the selected comorbidities are interlinked and how they influence each other's development and severity in the COVID-19 situation. Our proposed model is illustrated in Figures 1(a) and 1(b).

## 3. Results

### 3.1. Gene Expression Analysis

For investigating the identification and genomic association of COVID-19 comorbidities, we used RNA-seq datasets from NCBI. We selected DEGs eliminating duplicate dysregulated genes for COVID-19 and all other comorbidities, where upregulated DEGs were detected with a statistical *P* value < 0.05 and |logFC| > 1 and downregulated DEGs were considered with a *P* value < 0.05 and |logFC| < −1. Our analysis detected total 1164 DEGs for COVID-19, where 576 were upregulated and 588 were downregulated. For hypertension, we identified total 890 DEGs, where 745 DEGs were upregulated and 145 DEGs were downregulated. For diabetes, we got total 897 DEGs, where 417 showed upregulation and the rest 480 showed downregulation. In obesity, we found 997 DEGs, of which 510 were upregulated, and 487 were downregulated. Similarly, in lung cancer, total 4759 genes were differentially expressed with 2504 upregulated DEGs, and 2255 downregulated DEGs. The summary of the DEGs is shown in Table 2.

### 3.2. Comorbidity Identification

After doing all the computational and statistical analyses stated in the methods and analyses section, we picked top 50 diseases from the list of 7,092 diseases and removed homogeneous diseases. As a result, we obtained 27 divergent diseases which are breast cancer, liver cancer, colorectal cancer, prostate cancer, stomach cancer, lung cancer, melanoma, rheumatoid arthritis, ovarian cancer, tumor progression, glioma, Alzheimer's disease, obesity, asthma, glioblastoma, pancreatic cancer, atherosclerosis, hypertension, leukemia, diabetes mellitus, schizophrenia, neuroblastoma, degenerative polyarthritis, renal cell cancer, multiple sclerosis, systemic lupus erythematosus, and multiple myeloma, where hypertension, diabetes, obesity, and lung cancer are selected as comorbidities for further analysis. The selection criteria for the comorbidities are discussed in the methodology section.  Table 3 shows our resulted illness and comorbidities.

### 3.3. Comorbidity Association and Most Associated Comorbidity Identification

**Figure 2:**
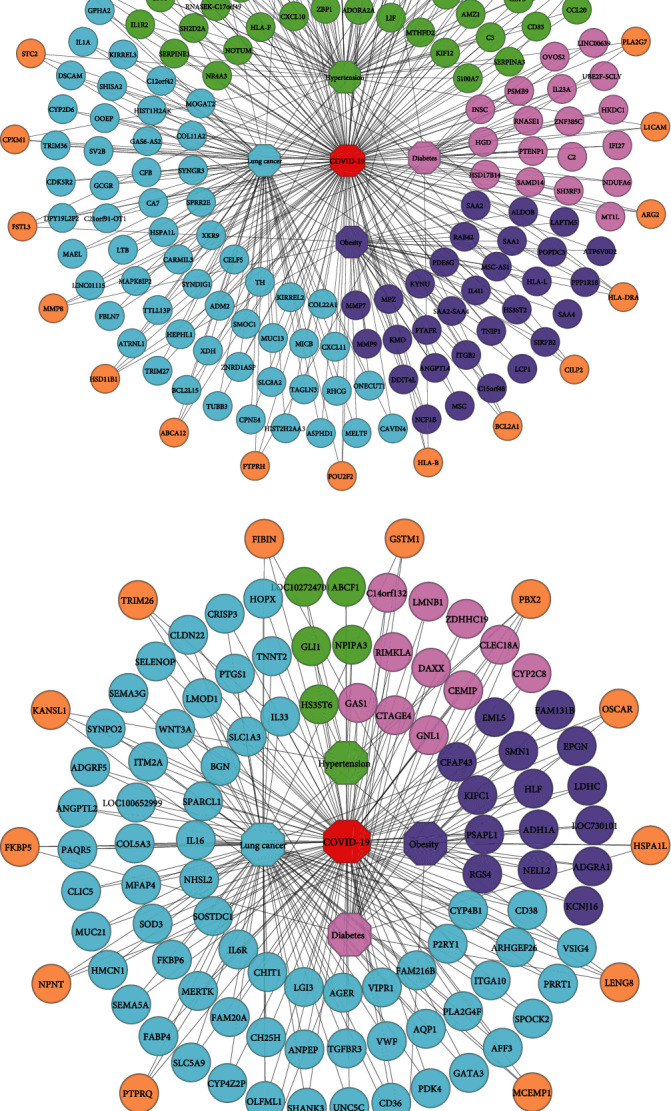
COVID-19 and comorbidity association networks for the upregulated and downregulated shared DEGs. (a) Association network for the upregulated shared DEGs. (b) Association network for the downregulated shared DEGs. The node legends are octagonal-shaped nodes for diseases and round-shaped nodes for DEGs. Green color nodes are shared between hypertension and COVID-19, pink color nodes are shared between diabetes and COVID-19, lavender color nodes are shared between obesity and COVID-19, and cyan color nodes are shared between lung cancer and COVID-19. Again, there is a shared pattern of orange color nodes between COVID-19 and several comorbidities. The edges or links indicate the relationship between the diseases and the DEGs.

To prove the associativity, there must have at least one common gene between the two maladies. Therefore, we performed cross-comparative analyses for the shared DEGs identification among COVID-19 and its selected comorbidities, where identified shared DEGs are considered to impact COVID-19 severity. Our findings demonstrated that COVID-19 shared total 78 (70 up and 08 down), 48 (27 up and 21 down), 63 (41 up and 22 down), and 144 (71 up and 73 down) DEGs with hypertension, diabetes, obesity, and lung cancer. In the upregulated shared DEGs, 55 adjacent DEGs are shared only between COVID-19 and hypertension, and these are closely located and shown in Figure 2(a). There are 15 DEGs more which are shared between COVID-19 and hypertension as well as other comorbidities. Similarly, diabetes and COVID-19 have 18 closely adjacent DEGs, and another 9 DEGs are shared among COVID-19, diabetes, and others. 30 DEGs are shared between obesity and COVID-19, and 11 DEGs are shared between COVID-19, obesity, and other comorbidities. The study also found that 57 DEGs are adjacently located between COVID-19 and lung cancer, and 14 DEGs are located between lung cancer, COVID-19, and others. In the downregulated shared DEGs, 5 adjacent DEGs are common only between hypertension and COVID-19; 3 DEGs are in between COVID-19 and others, including hypertension. Diabetes and COVID-19 have 11 DEGs in common, with another 10 DEGs between COVID-19, diabetes, and other comorbidities as shown in Figure 2(b). COVID-19 and obesity shared 15 DEGs; another 7 DEGs are shared between obesity and others with COVID-19. Finally, 62 DEGs are adjacently located and shared between COVID-19 and lung cancer, with 11 other DEGs among COVID-19, lung cancer, and others.

We also calculated the Jaccard similarity index utilizing the DEGs (upregulated and downregulated) of COVID-19 and hypertension as 0.039, COVID-19 and diabetes as 0.024, COVID-19 and obesity as 0.030, and COVID-19 and lung cancer as 0.025. The interaction between two nodes can be measured using neighborhood similarity (Jaccard's similarity) [38]. The higher the neighborhood similarity of the adjacent nodes, the more interaction between the two nodes [28, 39]. Among the four comorbidities, hypertension exhibited the highest Jaccard similarity score. In Table 4, the Jaccard similarity index is calculated using the DEGs of COVID-19 and the comorbidities. To exert the significant affinity, COVID-19 and comorbidity association networks (CCAN) are presented in Figure 2, where recurrent up and downregulated DEGs are used between the comorbidities and COVID-19.

### 3.4. Pathway and Ontology Enrichment Analyses

Utilizing molecular pathway and gene ontology enrichment analyses, we investigated the effective pathways and ontologies that have a direct or indirect association with the progression of COVID-19 comorbidities. The pathway enrichment analysis identified the pathways, “Immune System,” “Interferon Gamma signaling,” “T cell receptor signaling,” “Apoptosis,” “Tryptophan metabolism,” “Glycolysis/Gluconeogenesis,” “Matrix Metalloproteinases,” “Cytokine-cytokine receptor interaction,” “ECM-receptor interaction,” and “Protein digestion and absorption.” Again, “Inflammatory response,” “Response to lipopolysaccharide,” “Cytokine-mediated signaling pathway,” “Neutrophil degranulation,” “Neutrophil activation involved in immune response,” “Extracellular matrix organization,” “Extracellular structure organization,” “Integral component of plasma membrane,” “Collagen-containing extracellular matrix,” and “Synapse organization” are identified from ontology analysis based on the parameters described in the methodology section. Identified pathways and ontologies are shown in Table 5 and Table 6.

### 3.5. Protein-Protein Interaction (PPI) Analyses and Hub Protein Identification

**Figure 3:**
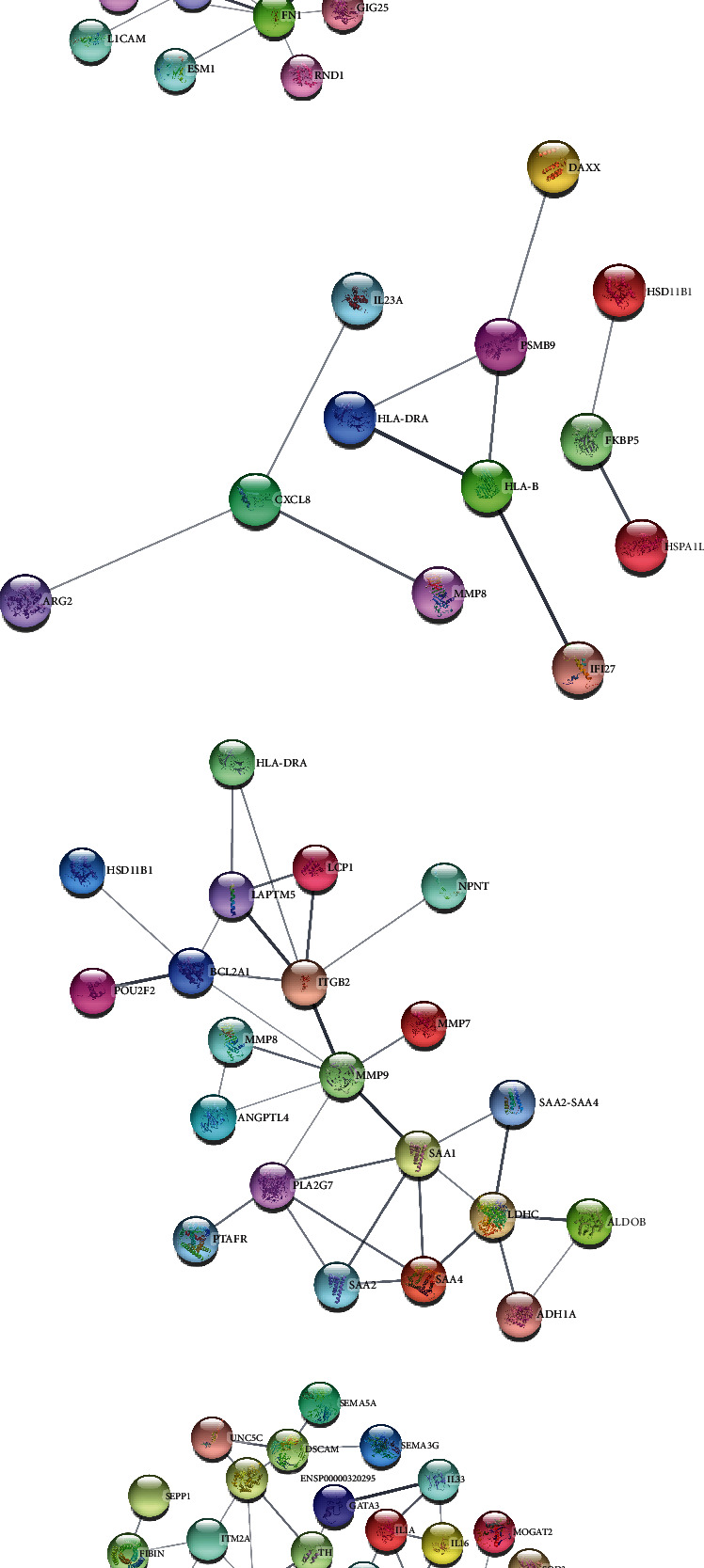
Protein-protein interaction networks. (a) PPI between COVID-19 and hypertension. (b) PPI between COVID-19 and diabetes. (c) PPI between COVID-19 and obesity. (d) PPI between COVID-19 and lung cancer. In the networks, the nodes denote proteins, and the edges denote interactions among the proteins.

To understand cell physiology and the diseases resulting from abnormal PPIs, we investigated PPI analyses. We got highly interacting proteins by removing the disconnected proteins from the PPI analyses.  Figure 3(a) depicts the PPI network between COVID-19 and hypertension. In the network, the number of proteins and edges is 44 and 141, and the PPI enrichment *P* value is < 1.0E-16.  Figure 3(b) depicts the PPI network between COVID-19 and diabetes, where the number of proteins and edges is 12 and 10, and the enrichment *P* value is 0.0332.  Figure 3(c) depicts the PPI network between COVID-19 and obesity. In that network, the number of proteins is 21, the number of edges is 32, and the enrichment *P* value is 1.5E-08.  Figure 3(d) depicts the PPI of COVID-19 and lung cancer. The number of proteins, edges, and enrichment *P* value is 68, 78, and 6.85E-08.  Figure 3 shows the PPI analyses between COVID-19 and each comorbidity.

**Figure 4:**
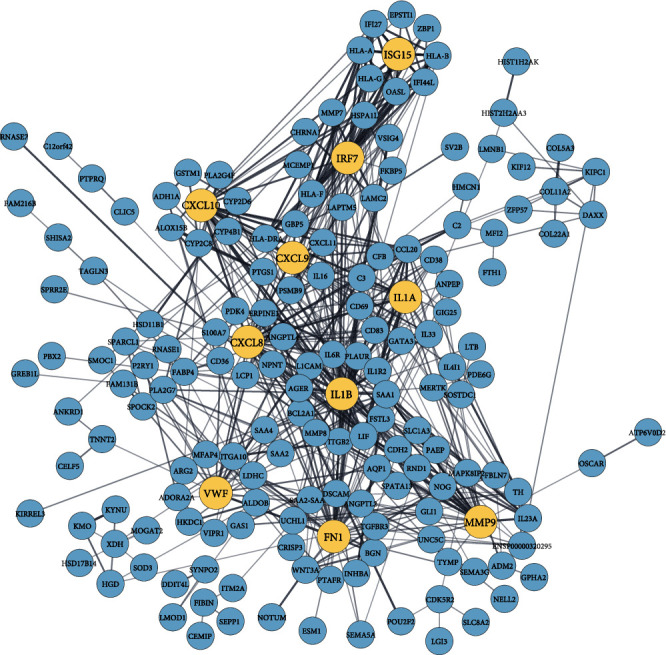
Hub proteins from PPI analysis, where the cyan color nodes are proteins and relatively large yellow color nodes are hub proteins.

By analyzing the PPI network derived from the overlapping DEGs, we identified 10 significant biomarker hub proteins based on the degree centrality using the cytoHubba module. In the network, there are 174 proteins and 545 edges, where hub proteins are marked in a different color. The enrichment *P* value for the network is < 1.0E-16. Our identified hub proteins are ranked according to their degrees as follows: IL1B (degree: 50), CXCL8 (degree: 38), FN1 (degree: 37), MMP9 (degree: 35), CXCL10 (degree: 32), IL1A (degree: 21), IRF7 (degree: 20), VWF (degree: 19), CXCL9 (degree: 19), and ISG15 (degree: 18). Identified biomarker hub proteins are shown in Figure 4.

### 3.6. Intercomorbidity Analysis

**Figure 5:**
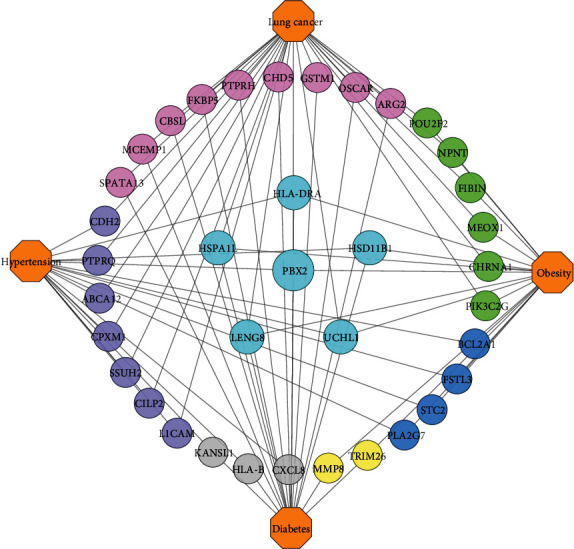
Intercomorbidity association network relating the comorbidities together through the linking DEGs where the octagonal-shaped nodes are the comorbidities, while the round-shaped nodes are the identified significant linking DEGs. Pink nodes connected diabetes and lung cancer; green nodes connected obesity and lung cancer; yellow nodes connected diabetes and obesity; gray nodes connected hypertension and diabetes, and lavender color nodes connected hypertension with lung cancer. The relatively large cyan color nodes interconnected more than 2 comorbidities, whereas PBX2 conjoined all of the four comorbidities.

A further observation is that our final comorbidities are interacted with each other by exchanging the linking DEGs among themselves. Besides, their interaction, evolvement, and development are also documented in international publications. We ascertained that hypertension, diabetes, obesity, and lung cancer shared 1 DEG; hypertension, diabetes, and obesity shared 2 DEGs; hypertension, obesity, and lung cancer shared 1 DEG; diabetes, obesity, and lung cancer shared 2 DEGs. Again, hypertension and diabetes shared 3 DEGs; obesity and diabetes shared 2 DEGs; hypertension and obesity shared 4 DEGs; also, obesity and lung cancer shared 6 DEGs. Finally, hypertension and lung cancer as well as diabetes and lung cancer shared 7 and 9 DEGs.  Figure 5. shows an association network to illustrate the interaction among the comorbidities.

## 4. Discussion

To determine a disease's comorbidities, several methods and analyses have been developed, including clinical tests and meta-analyses. But the procedures are difficult, exhausting, and time consuming. Therefore, our research is aimed at developing a bioinformatics and system biological framework to identify comorbidities and provide genetic and physiological insights related to COVID-19 along with their interrelationship.

Based on clinical and etiological evidence, we found that our identified diseases, breast cancer [40], liver cancer [41], colorectal cancer [42], prostate cancer [43], stomach cancer [44], lung cancer [45], melanoma [46], rheumatoid arthritis [47], ovarian cancer [48], tumor progression [49], glioma [50], Alzheimer's disease [51], obesity [52], asthma [53], glioblastoma [54], pancreatic cancer [55], atherosclerosis [56], hypertension [52], leukemia [57], diabetes mellitus [52], schizophrenia [58], neuroblastoma [59], degenerative polyarthritis [60], renal cell cancer [61], multiple sclerosis [33], systemic lupus erythematosus [62], and multiple myeloma [63], are related to COVID-19. Furthermore, we noted that hypertension, diabetes, obesity, and lung cancer are conditions that can cause COVID-19 to progress more severely [3, 52, 64]; therefore, we evaluated them as COVID-19 comorbidities.

We identified significant overlapping DEGs among COVID-19 and selected comorbidities which verify the susceptibility of the comorbidities to COVID-19 progression and severity. Our distinct (CCAN) networks for the up and downregulated genes indicate that COVID-19 is linked to hypertension, diabetes, obesity, and lung cancer in Figure 2. Accordingly, we calculated the Jaccard similarity index for the comorbidities using both up and downregulated DEGs along with the shared DEGs shown in Table 4, where the highest Jaccard similarity value was found for hypertension, followed by obesity, lung cancer, and diabetes. Since hypertension has the highest index value, we propose that COVID-19 has a significant interaction with hypertension, and it is the most correlated comorbidity across all four COVID-19 comorbidities.

In order to understand molecular mechanisms underlying COVID-19-associated comorbidities, pathway and ontology enrichments were investigated using shared DEGs. In our identified pathways, the “Immune System” is prominent in the development of hypertension [65]. The “interferon gamma signaling” pathway modulates immune responses as well as influences hypertension [66]. The “T cell receptor signaling” and “Apoptosis” pathways are involved in diabetes progression [67, 68]. The pathways “Tryptophan metabolism” and “Glycolysis/Gluconeogenesis” are associated with obesity pathophysiology [69]. Similarly, the “Matrix Metalloproteinases” play a potential role in obesity as an activator and inhibitor [70]. The pathways “Cytokine-cytokine receptor interaction” and “ECM-receptor interaction” are found in lung cancer prognosis [71, 72]. And “Protein digestion and absorption” acts as a significant pathway in lung cancer development [73]. Again, in our identified ontologies, “Inflammatory response” is a significant causal ontology leading to hypertension [74]. The ontology “Response to lipopolysaccharide” is involved in hypertension [75]. Gene ontology, “Cytokine-mediated signaling pathway,” “Neutrophil degranulation,” and “Neutrophil activation involved in immune response,” are associated with and impact diabetes [76–78]. The “Extracellular matrix organization” as well as “Extracellular structure organization” ontologies have implications for the pathogenesis of obesity and metabolic dysfunction [79]. The “Integral component of plasma membrane” and “Collagen-containing extracellular matrix” ontologies are responsible for lung cancer prognosis [80–84]. Further, the ontology “Synapse organization” participates and plays a crucial role in lung cancer advancement. [85]. The summary of the molecular pathways and gene ontologies is presented in Table 5 and Table 6.

Based on COVID-19 shared DEGs, we identified 10 highly connected biomarker hub proteins that are responsible for the emergence of selected comorbidities. We validated the emergence by literature review and found a strong affiliation between the hub proteins and the continuation of comorbidities. Interleukin 1 beta (IL-1B) is a member of the interleukin 1 cytokine family that increases hypertension and negatively impacts end-organs during high blood pressure [86]. IL-1B is also involved in the genetic background of diabetes, obesity, and lung cancer prognosis [87–89]. C-X-C motif chemokine ligand 8 (CXCL8) is a major mediator of the inflammatory response that is significantly associated with and susceptible to hypertension, and diabetes has a positive correlation with obesity and lung cancer [90–93]. Fibronectin 1 (FN1) is a member of the glycoprotein family, which is involved and functions as a biomarker in hypertension, diabetes, obesity, and lung cancer [94–96]. Matrix metallopeptidase 9 (MMP9) is a matrixin that is involved in the degradation of the extracellular matrix as well as associated with hypertension, diabetes, obesity, and lung cancer as a marker gene [97–100]. C-X-C motif chemokine 10 (CXCL10) is a small cytokine belonging to the CXC chemokine family that is correlated and responsible for the greater susceptibility of patients with severe portal hypertension, diabetes, obesity, and lung cancer [101–104]. Interleukin 1 alpha (IL1A) is a member of the interleukin 1 cytokine family that is involved in the molecular mechanisms of hypertension, diabetes, obesity, and lung cancer [88, 105–107]. Interferon regulatory factor 7 (IRF7) is a member of the interferon regulatory transcription factor family and is involved in the pathogenesis of diabetes, obesity, and lung cancer [108–110]. Von Willebrand factor (VWF) is a blood glycoprotein, and elevated VWF is a biomarker of hypertension, diabetes, obesity, and lung cancer [83, 111–113]. C-X-C motif chemokine ligand 9 (CXCL9) is a small cytokine belonging to the CXC chemokine family, and it is associated with hypertension, diabetes, obesity, and lung cancer [114–117]. As well, interferon-stimulated gene 15 (ISG15) is induced by type I interferon and is correlated with hypertension and lung cancer [118–120]. The summary of the biomarker hub proteins is presented in Table 7.

Again, our identified significant linking DEGs linked the comorbidities together. We found that PBX2 linked hypertension, diabetes, obesity, and lung cancer with each other. Additionally, HSD11B1 and HLA-DRA linked hypertension, diabetes, and obesity; UCHL1 linked hypertension, obesity, and lung cancer; LENG8 and HSPA1L linked diabetes, obesity, and lung cancer. Besides, hypertension and diabetes are linked by CXCL8, HLA-B, and KANSL1; diabetes and obesity are linked by TRIM26 and MMP8; hypertension and obesity are linked by PLA2G7, FSTL3, STC2, and BCL2A1. Furthermore, PIK3C2G, FIBIN, CHRNA1, NPNT, MEOX1, and POU2F2 linked obesity and lung cancer, while PTPRQ, SSUH2, CILP2, CDH2, ABCA12, CPXM1, and L1CAM linked hypertension and lung cancer. Finally, we found ARG2, PTPRH, CBSL, GSTM1, FKBP5, MCEMP1, OSCAR, CHD5, and SPATA13, which linked diabetes and lung cancer. Public evidence also supports our findings and suggests that hypertension and diabetes have pathophysiological links [121]; diabetes and obesity are metabolically connected [122]; obesity is closely associated with hypertension [123]; diabetes and obesity are related to cancer progression [124]; hypertension and diabetes are cancer risk factors [125]; diabetes and lung cancer are correlated [126]; hypertension, obesity, and type 2 diabetes are associated with one another [127], and a complex interrelationship exists between obesity, diabetes, and cancers [128].  Table 8 summarizes details of identified significant linking DEGs among the comorbidities in terms of gene symbol, gene name, gene type, gene expression, and a brief description.

The above discussion implies that our identified results from the COVID-19 dataset have a significant association and influence on hypertension, diabetes, obesity, and lung cancer progression. Consequently, we conclude that they are COVID-19 comorbidities, and our framework is valid for comorbidity detection and evaluation.

## 5. Conclusion

COVID-19 comorbidities increase disease severity and mortality as well as affect organ damage. Therefore, it is crucial for healthcare practitioners to develop an approach to identify comorbidities and explore their biomolecular mechanisms in order to come up with effective therapeutics for COVID-19. In this study, we explain how our methodology identifies COVID-19 comorbidities, explores genomic profiles, and highlights molecular insights and checkpoints related to potential biomarkers such as DEGs, pathways, ontologies, PPIs, hub proteins, and prognostic features relevant to COVID-19. However, it is still necessary to conduct in vitro and in vivo experiments. In fine, we suggest that our model can assist in comorbidity and biomolecular process diagnosis early of other diseases, if transcriptomic datasets are available, and thus, this model can minimize the financial burden on healthcare systems.

## Figures and Tables

**Table 1:** Dataset description for COVID-19 and selected comorbidities.

Disease name	GEO number	Organism	Tissue	Sample type	Platform ID	Platform	Reference
SARS-CoV-2 (COVID-19)	GSE150819	Homo sapiens	Bronchial epithelial cells	SRA	GPL24676	Illumina NovaSeq 6000	[19]
Hypertension	GSE143953	Homo sapiens	Placenta cells	SRA	GPL20301	Illumina HiSeq 4000	[20]
Diabetes	GSE136053	Homo sapiens	Blood mononuclear cells	SRA	GPL16791	Illumina HiSeq 2500	[21]
Obesity	GSE55008	Homo sapiens	Omental adipose tissues	SRA	GPL11154	Illumina HiSeq 2000	[22]
Lung cancer	GSE60052	Homo sapiens	Lung tissues	SRA	GPL11154	Illumina HiSeq 2000	[23]

**Table 2:** Identified DEGs along with upregulated, downregulated, and overlapped DEGs for COVID-19 and its comorbidities.

Disease name	GSE number	Raw genes	Differentially expressed genes (DEGs)	Upregulated DEGs	Downregulated DEGs	Overlapping DEGs with COVID-19	
						Upregulated	Downregulated
SARS-CoV-2 (COVID-19)	GSE150819	20893	1164	576	588	—	—
Hypertension	GSE143953	19558	890	745	145	70	08
Diabetes	GSE136053	16972	897	417	480	27	21
Obesity	GSE55008	2211	997	510	487	41	22
Lung cancer	GSE60052	26938	4759	2504	2255	71	73

**Table 3:** Identified COVID-19 correlated illness and comorbidities.

Serial no.	Comorbidity name	*P* value	Gene counts
1	Breast cancer	6.74*E* − 03	324
2	Liver cancer	7.24*E* − 07	272
3	Colorectal cancer	7.08*E* − 07	253
4	Prostate cancer	9.67*E* − 05	229
5	Stomach cancer	1.87*E* − 09	205
6	Lung cancer	6.86*E* − 07	199
7	Melanoma	9.58*E* − 06	191
8	Rheumatoid arthritis	5.95*E* − 16	190
9	Ovarian cancer	3.61*E* − 08	187
10	Tumor progression	1.48*E* − 08	181
11	Glioma	7.46*E* − 07	181
12	Alzheimer's disease	4.06*E* − 05	156
13	Obesity	2.25*E* − 04	150
14	Asthma	1.88*E* − 15	148
15	Glioblastoma	2.62*E* − 04	148
16	Pancreatic cancer	7.20*E* − 05	147
17	Atherosclerosis	9.50*E* − 19	142
18	Hypertensive disease (hypertension)	1.58*E* − 12	139
19	Leukemia	1.41*E* − 02	135
20	Diabetes mellitus	3.40*E* − 07	134
21	Schizophrenia	2.21*E* − 02	132
22	Neuroblastoma	5.85*E* − 04	130
23	Degenerative polyarthritis	1.36*E* − 18	128
24	Renal cell cancer	1.61*E* − 07	124
25	Multiple sclerosis	1.50*E* − 12	123
26	Systemic lupus erythematosus	1.06*E* − 11	121
27	Multiple myeloma	4.90*E* − 06	115

**Table 4:** Calculation of the Jaccard similarity index to understand the interaction between COVID-19 and each of the comorbidities. *T*_*C*19_ is the DEGs for COVID-19, where TU_*C*19_ is the upregulated DEGs and TD_*C*19_ is the downregulated DEGs. Similarly, *T*_CO_ corresponds to the DEGs for each comorbidity, where TU_CO_ is the upregulated and TD_CO_ is the downregulated DEGs.

Comorbidity name	TU_*C*19_	TD_*C*19_	*T*_*C*19_ = TU_*C*19_ + TD_*C*19_	TU_CO_	TD_CO_	*T*_CO_ = TU_CO_ + TD_CO_	*T*_*C*19_∩*T*_CO_	*T*_*C*19_ ∪ *T*_CO_ = *T*_*C*19_ + *T*_CO_ − (*T*_*C*19_∩*T*_CO_)	Jaccard's similarity index $\frac{\left|T_{C19}\cap T_{\text{CO}}\right|}{\;\left|T_{C19}\cup T_{\text{CO}}\right|}$
Hypertension	576	588	1164	745	145	890	78	1976	$\frac{78}{1976}=0.039$
Diabetes				417	480	897	48	2013	$\frac{48}{2013}=0.024$
Obesity				510	487	997	63	2098	$\frac{63}{2098}=0.030$
Lung cancer				2504	2255	4759	144	5779	$\frac{144}{5779}=0.025$

**Table 5:** Identified significant molecular pathways associated with the comorbidities.

Database	Molecular pathway	*P* value	Genes in the pathway	Associated comorbidity
WikiPathway	Immune system	7.80*E* − 05	ZBP1; DUSP5; GBP5; RASGRF2; IL1R2; HLA-B; LIF; FN1; ISG15; HLA-A; HLA-F; HLA-G; OASL; C3; IL1B; IRF7; HLA-DRA	Hypertension
Reactome	Interferon gamma signaling	2.89*E* − 09	GBP5; HLA-B; IRF7; HLA-DRA; HLA-A; HLA-F; HLA-G; OASL	Hypertension
Reactome	T cell receptor signaling	3.26*E* − 02	HLA-DRA; PSMB9	Diabetes
KEGG	Apoptosis	4.57*E* − 02	DAXX; LMNB1	Diabetes
KEGG	Tryptophan metabolism	3.13*E* − 04	IL4I1; KYNU; KMO	Obesity
KEGG	Glycolysis/gluconeogenesis	1.24*E* − 03	LDHC; ADH1A; ALDOB	Obesity
WikiPathway	Matrix metalloproteinases	1.14*E* − 04	MMP7; MMP8; MMP9	Obesity
KEGG	Cytokine-cytokine receptor interaction	2.01*E* − 02	IL1A; IL33; CXCL11; IL16; LTB; IL6R	Lung cancer
KEEG	ECM-receptor interaction	4.38*E* − 04	VWF; SV2B; ITGA10; CD36; NPNT	Lung cancer
KEEG	Protein digestion and absorption	6.57*E* − 03	COL5A3; COL22A1; COL11A2; SLC8A2	Lung cancer

**Table 6:** Identified significant gene ontologies associated with the comorbidities.

Database	GO ID	Gene ontology	*P* value	Genes in the ontology	Associated comorbidity
GO biological process	GO: 0006954	Inflammatory response	3.18*E* − 06	SERPINA3; CXCL10; CXCL9; CXCL8; ADORA2A; CCL20; IL1B; ABCF1	Hypertension
GO biological process	GO: 0032496	Response to lipopolysaccharide	2.89*E* − 06	CXCL10; CXCL9; CXCL8; IL1B; SERPINE1; ANKRD1; S100A7	Hypertension
GO biological process	GO: 0019221	Cytokine-mediated signaling pathway	1.04*E* − 04	CXCL8; IFI27; IL23A; HLA-B; HLA-DRA; TRIM26; LMNB1; PSMB9	Diabetes
GO biological process	GO: 0043312	Neutrophil degranulation	2.80*E* − 02	HLA-B; MCEMP1; MMP8; OSCAR	Diabetes
GO biological process	GO: 0002283	Neutrophil activation involved in immune response	2.87*E* − 02	HLA-B; MCEMP1; MMP8; OSCAR	Diabetes
GO biological process	GO: 0030198	Extracellular matrix organization	3.59*E* − 04	MMP7; ITGB2; LCP1; MMP8; NPNT; MMP9	Obesity
GO biological process	GO: 0043062	Extracellular structure organization	5.93*E* − 04	MMP7; ITGB2; MMP8; NPNT; MMP9	Obesity
GO cellular component	GO: 0005887	Integral component of plasma membrane	8.31*E* − 03	KIRREL3; CHRNA1; VIPR1; KIRREL2; GCGR; SEMA3G; SLC1A3; PTPRH; MERTK; AGER; SLC8A2; AQP1; TGFBR3; CDH2; P2RY1; SYNDIG1; CD36; RHCG; IL6R	Lung cancer
GO cellular component	GO: 0062023	Collagen-containing extracellular matrix	3.82*E* − 06	VWF; ATRNL1; COL11A2; BGN; NPNT; L1CAM; SOD3; MFAP4; CDH2; COL5A3; SMOC1; ANGPTL2; HMCN1	Lung cancer
GO biological process	GO: 0050808	Synapse organization	3.65*E* − 06	KIRREL3; CHRNA1; CDH2; DSCAM; SPOCK2; L1CAM; SHANK3; SLC8A2	Lung cancer

**Table 7:** Identified significant biomarker hub proteins in the continuation of the comorbidities.

Rank	Symbol	Name	Degree	Affiliated comorbidity
1	IL1B	Interleukin 1 beta	50	Hypertension, diabetes, obesity, and lung cancer.
2	CXCL8	C-X-C motif chemokine ligand 8	38	Hypertension, diabetes, obesity, and lung cancer.
3	FN1	Fibronectin 1	37	Hypertension, diabetes, obesity, and lung cancer.
4	MMP9	Matrix metallopeptidase 9	35	Hypertension, diabetes, obesity, and lung cancer.
5	CXCL10	C-X-C motif chemokine 10	32	Hypertension, diabetes, obesity, and lung cancer.
6	IL1A	Interleukin 1 alpha	21	Hypertension, diabetes, obesity, and lung cancer.
7	IRF7	Interferon regulatory factor 7	20	Diabetes, obesity, and lung cancer.
8	VWF	Von Willebrand factor	19	Hypertension, diabetes, obesity, and lung cancer.
8	CXCL9	C-X-C motif chemokine ligand 9	19	Hypertension, diabetes, obesity, and lung cancer.
10	ISG15	Interferon-stimulated gene 15	18	Hypertension and lung cancer.

**Table 8:** List of linking DEGs with gene expression pattern and synopsis involved in the intercomorbidity association.

Symbol	Name	Gene type	Gene expression	Synopsis	Reference
PBX2	PBX homeobox 2	Protein coding	Downregulated	PBX2 is a member of the TALE/PBX homeobox family that regulates TLX1 promoter translocation in acute pre-B-cell leukemias.	[129]
HSD11B1	Hydroxysteroid 11-beta dehydrogenase 1	Protein coding	Upregulated	HSD11B1 encodes a microsomal enzyme that catalyzes the conversion of cortisone to cortisol and stress hormone cortisol. A high level of cortisol can result in obesity.	[130]
HLA-DRA	Major histocompatibility complex, class II, DR alpha	Protein coding	Upregulated	The HLA class II molecule HLA-DRA plays a key role in the immune system and responses to pathogen-derived peptides.	[131]
UCHL1	Ubiquitin C-terminal hydrolase L1	Protein coding	Upregulated	In neurons and diffuse neuroendocrine system cells, the UCHL1 gene is expressed. Mutations in this gene are associated with Parkinson's disease.	[132]
LENG8	Leukocyte receptor cluster member 8	Protein coding	Downregulated	Among the tissues, the LENG8 gene is expressed in the spleen, endometrium, testis, small intestine, and other areas and is predicted to be functional in the nucleus.	[133]
HSPA1L	Heat shock protein family A (Hsp70) member 1 like	Protein coding	Downregulated	HSPA1L is found in the histocompatibility complex class III region and encodes a 70 kDa heat shock protein. Together with other heat shock proteins, it stabilizes existing proteins.	[134]
CXCL8	C-X-C motif chemokine ligand 8	Protein coding	Upregulated	CXCL8 encodes a protein that is a member of the CXC chemokine family and is referred to as interleukin-8 (IL-8). It is a major mediator of the inflammatory response and plays a role in the proinflammatory signaling cascade, systemic inflammatory response syndrome, lower respiratory tract infection bronchiolitis, lung inflammation, coronary artery disease, endothelial dysfunction, and metastasis.	[135]
HLA-B	Major histocompatibility complex, class I, B	Protein coding	Upregulated	HLA-B is a member of the HLA class I which plays a vital role by presenting peptides derived from the endoplasmic reticulum lumen in the immune system.	[136]
KANSL1	KAT8 regulatory NSL complex subunit 1	Protein coding	Downregulated	The nuclear protein KANSL1 is expressed in the brain, testis, kidney, ovary, thyroid, and other tissues. Moreover, it is a part of two protein complexes including histone acetylation, the NSL1 complex and the MLL1 complex.	[137]
TRIM26	Tripartite motif containing 26	Protein coding	Downregulated	TRIM26 belongs to the tripartite motif (TRIM) family and is found in cytoplasmic bodies. In addition, DNA binding is assumed to be a function of this protein.	[138]
MMP8	Matrix metallopeptidase 8	Protein coding	Upregulated	MMP8 is a member of the matrix metalloproteinase (MMP) family, which is involved in embryonic development, reproduction, tissue remodeling, and disease processes (arthritis and metastasis).	[139]
PLA2G7	Phospholipase A2 group VII	Protein coding	Upregulated	The protein PLA2G7 is a secreted enzyme that catalyzes and causes the degradation of platelet-activating factors into biological inactive products.	[140]
FSTL3	Follistatin like 3	Protein coding	Upregulated	FSTL3 is a glycoprotein in the follistatin-module-protein family that may promote leukemogenesis.	[141]
STC2	Stanniocalcin 2	Protein coding	Upregulated	Homodimeric glycoprotein STC2 is expressed in various tissues and may play a role in regulating renal and intestinal calcium, phosphate transport, cell metabolism, cellular calcium/phosphate homeostasis, and autocrine or paracrine functions.	[142]
BCL2A1	BCL2 related protein A1	Protein coding	Upregulated	BCL2A1 is a member of the BCL-2 protein family that forms heterodimers and homodimers and is involved in cellular activities like embryonic development, homeostasis, and tumorigenesis.	[143]
PIK3C2G	Phosphatidylinositol-4-phosphate 3-kinase catalytic subunit type 2 gamma	Protein coding	Downregulated	The protein PIK3C2G belongs to the phosphoinositide 3-kinase (PI3K) family. PI3-kinases are involved in protein-protein interactions, several diseases (type II diabetes), and signaling pathways including cell proliferation, oncogenic transformation, cell survival, cell migration, and intracellular protein trafficking.	[144]
FIBIN	Fin bud initiation factor homolog	Protein coding	Downregulated	FIBIN can be found in the Golgi apparatus, endoplasmic reticulum, or extracellular region. It activates protein homodimerization and protein kinase C signaling as well as responses to dexamethasone and manganese ions.	[145]
CHRNA1	Cholinergic receptor nicotinic alpha 1 subunit	Protein coding	Upregulated	The CHRNA1 gene is expressed in the prostate, colon, lymph nodes, and other tissues and participates in acetylcholine binding and channel gating.	[146]
NPNT	Nephronectin	Protein coding	Downregulated	NPNT is found in extracellular exosomes as well as in collagen-containing extracellular matrix. It is involved in several processes including cell-cell adhesion, positive regulation of osteoblasts, and the ERK1 and ERK2 cascade.	[147]
MEOX1	Mesenchyme homeobox 1	Protein coding	Upregulated	MEOX1 is expressed in fat, heart, spleen, urinary bladder, and other tissues that may influence the molecular signaling network.	[148]
POU2F2	POU class 2 homeobox 2	Protein coding	Upregulated	The POU2F2 gene encodes a homeobox protein of the POU domain family that binds the octamer transcription factor.	[149]
PTPRQ	Protein tyrosine phosphatase receptor type Q	Protein coding	Downregulated	PTPRQ locus is from the type III receptor-like protein-tyrosine phosphatase family that promotes dephosphorylation and phosphatidylinositol and influences cellular proliferation and differentiation.	[150]
SSUH2	Ssu-2 homolog	Protein coding	Upregulated	Its expression can be found in the testes, small intestines, duodenums, and cytoplasms as well as in the nucleus. Again, SSUH2 plays a role in odontogenesis.	[151]
CILP2	Cartilage intermediate layer protein 2	Protein coding	Upregulated	CILP2 is found in extracellular exosomes as well as in the testis, gall bladder, bone marrow, and other tissues.	[152]
CDH2	Cadherin 2	Protein coding	Upregulated	CDH2 is a classical cadherin protein that belongs to the cadherin superfamily. It contributes to the development of the nervous system, left-right asymmetry, cartilage, and bone structure.	[153]
ABCA12	ATP binding cassette subfamily A member 12	Protein coding	Upregulated	ABCA12 is a membrane-associated protein from the ATP-binding cassette (ABC) transporter family. Molecules are transferred within extra and intracellular membranes by ABC proteins.	[154]
CPXM1	Carboxypeptidase X, M14 family member 1	Protein coding	Upregulated	CPXM1 expresses in the endometrium, gall bladder, urinary bladder, and other tissues and encodes a protein of the carboxypeptidase family.	[155]
L1CAM	L1 cell adhesion molecule	Protein coding	Upregulated	The L1CAM axonal glycoprotein belongs to the immunoglobulin supergene family, which can be involved in the development of the nervous system, neural migration, and differentiation.	[156]
ARG2	Arginase 2	Protein coding	Upregulated	ARG2 is expressed in the thyroid, prostate, kidney, and other tissues that facilitate the hydrolysis of arginine into urea and amino acids.	[157]
PTPRH	Protein tyrosine phosphatase receptor type H	Protein coding	Upregulated	PTPRH is usually present in the brain and liver, cancer cells, and at lower levels in the heart and stomach, where it influences several cellular processes such as cell growth, differentiation, the mitotic cycle, and oncogenic transformation. It belongs to the protein tyrosine phosphatase (PTP) group.	[158]
CBSL	Cystathionine beta-synthase like	Protein coding	Upregulated	CBSL homotetramer catalyzes homocysteine to cystathionine conversion and involves in cellular H_2_S production. Deficiencies of this gene are responsible for cystathionine beta-synthase deficiency which causes homocystinuria.	[159, 160]
GSTM1	Glutathione S-transferase mu 1	Protein coding	Downregulated	GSTM1 belongs to the mu class that encodes a glutathione S-transferase as well as functions in carcinogens, therapeutic drugs, environmental toxins, and products of oxidative stress.	[161]
FKBP5	FKBP prolyl isomerase 5	Protein coding	Downregulated	FKBP5 is found in fat, lymph node, esophagus, and other tissues and belongs to the immunophilin protein family, which is involved in immunoregulation and fundamental cellular mechanisms like protein folding and trafficking.	[162]
MCEMP1	Mast cell expressed membrane protein 1	Protein coding	Downregulated	MCEMP1 is a single-pass transmembrane protein, which is biasedly expressed in the lung, bone marrow, appendix, and other tissues. It is hypothesized that MCEMP1 is involved in cell differentiation or immune responses.	[163]
OSCAR	Osteoclast-associated Ig-like receptor	Protein coding	Downregulated	The gene encodes the leukocyte receptor complex protein that is associated with osteoclasts. The gene is associated with bone homeostasis, innate and adaptive immune responses, oxidative stress-mediated atherogenesis, and monocyte adhesion.	[164]
CHD5	Chromodomain helicase DNA binding protein 5	Protein coding	Upregulated	CHD5 is a neuron-specific protein from the chromodomain helicase DNA-binding protein family that is responsible for neuroblastoma development. It may also involve in chromatin remodeling and gene transcription.	[165]
SPATA13	Spermatogenesis-associated 13	Protein coding	Downregulated	SPATA13 is involved in cell migration, migration regulation, plasma membrane-bound cell projection assembly, identical protein binding activity, and guanyl-nucleotide exchange activity. Meanwhile, it is expressed in the spleen, lymph node, appendix, kidney, and other tissues.	[166]

## Data Availability

The datasets are available in the public repository of the National Center for Biotechnology Information (NCBI) (https://www.ncbi.nlm.nih.gov/) under the accession numbers GSE150819, GSE143953, GSE136053, GSE55008, and GSE60052.
